# The ubiquitin-proteasome system in the tumor immune microenvironment: a key force in combination therapy

**DOI:** 10.3389/fimmu.2024.1436174

**Published:** 2024-09-09

**Authors:** Yongmei Wang, Saisai Li, Wenqin Wang

**Affiliations:** ^1^ Breast Disease Center, The Affiliated Hospital of Qingdao University, Qingdao, Shandong, China; ^2^ Department of Hematology, The Affiliated Hospital of Qingdao University, Qingdao, Shandong, China; ^3^ School of Life Sciences, Shandong University, Qingdao, Shandong, China

**Keywords:** UPS, TIME, immune cells, immunotherapy, cancer

## Abstract

The ubiquitin-proteasome system (UPS) plays a crucial role in modulating the proliferation, activation, and normal functioning of immune cells through the regulation of protein degradation and function. By influencing the expression of immune checkpoint-associated proteins, the UPS modulates T cell-mediated anti-tumor immune responses and can potentially facilitate the immune escape of tumor cells. Additionally, the UPS contributes to the remodeling of the tumor immunosuppressive microenvironment (TIME) by regulating B cells, dendritic cells (DCs), macrophages, and Treg cells. Targeting the UPS in conjunction with immune checkpoint-associated proteins, and combining these with other therapeutic approaches, may significantly enhance the efficacy of combination therapies and pave the way for novel cancer treatment strategies. In this review, we first summarize the composition and alterations of the TIME, with a particular emphasis on the role of the UPS in TIME and its interactions with various immune cell types. Finally, we explore the potential of combining UPS-targeted therapies with immunotherapy to substantially improve the effectiveness of immunotherapy and enhance patient survival outcomes.

## 1 Introduction

The UPS is a unique intracellular protein degradation mechanism that primarily labels proteins with highly conserved ubiquitin polypeptides, facilitating the recognition and degradation of target proteins by the 26S proteasome (1, 2). Over 20 years ago, researchers identified that intracellular proteins are conjugated to highly conserved small ubiquitin polypeptides, forming complexes that bind to the 26S proteasome and are ultimately recognized and degraded into small molecular compounds. As scientific understanding of the UPS’s structure and function has deepened, it has been revealed that ubiquitination is a process in which a series of ubiquitinating enzymes (E1, E2, and E3) sequentially transfer ubiquitin molecules to specific intracellular targets (3). In this process, E1 first activates the ubiquitin molecule through ATP hydrolysis and uses the released energy to generate a unique ubiquitin (Ub)-E1 complex. The E1 complex is then transferred to a cysteine residue at the E2 active site (4). Assisted by E3 ubiquitin ligase, the E2 complex facilitates the conjugation of ubiquitin to the substrate, thereby ensuring substrate specificity (5). The typical ubiquitination process involves the C-terminal glycine of ubiquitin (Glycine 76) actively recognizing and binding to lysine residues of the target protein, forming an isopeptide bond that subsequently affects normal protein degradation. Atypical ubiquitination, such as that formed through ester or thioester bonds, while less common, has also demonstrated unique biological significance in regulating certain cellular processes (6). Moreover, ubiquitin itself has eight potential ubiquitination sites, including K6, K11, K27, K33, K29, M1, K48, and K63. This allows ubiquitin to form various ubiquitin chains, further adding to the complexity and regulation of the ubiquitination process (7).

Cancer is an extremely complex disease, involving a combination of processes such as genetic mutations, DNA damage, immune escape, and aging. The high expression of tumor immune checkpoint proteins inhibits immune recognition and normal immune responses (8). In particular, programmed death receptor 1 (*PD-1*) and programmed death ligand 1 (*PD-L1)* significantly suppress immune responses in the TME by interacting with T cells (9), thereby promoting tumor proliferation and metastasis (10). The UPS plays a critical role in regulating PD-1/PD-L1 expression and function, providing novel regulatory targets for cancer immunotherapy (11).

This review aims to summarize the intricate relationship between the UPS and the TIME. The focus is on detailing the interactions between the UPS and various immune cell types, particularly the alterations in the PD-1 pathway in T cells. By exploring the mechanisms of UPS action in TIME and its role in PD-1/PD-L1 expression and stability, we can better leverage these targets to develop novel therapeutic strategies, enhancing the efficacy of cancer treatment and improving patient survival outcomes.

## 2 Composition and function of immune cells in the TIME

In the TIME, various immune cells interact to form a complex network comprising both anti-tumor immune cells, which can attack tumor cells, and pro-tumor immune cells, which promote tumor proliferation and spread. The homeostatic balance of these cells is crucial in regulating tumor progression and response to therapy.

### 2.1 Anti-tumor immune cells in TIME

Anti-tumor immune cells are well-known for their ability to kill tumor cells through complex regulatory mechanisms. These cells include cytotoxic T lymphocytes (CTLs), natural killer (NK) cells, classically activated macrophages (M1 macrophages), and helper T cells (Th cells) (12). However, within the TIME, the normal activation and function of these anti-tumor immune cells can be significantly inhibited.

With the advancement of single-cell sequencing technology, the roles of T cells in anti-tumor immune responses differ across various subtypes (13). Naive T cells: Naive T cells themselves are not directly anti-tumor cells, but they can differentiate into effector T cells upon antigen stimulation, thereby participating in the anti-tumor immune response. Effector T cells: Effector T cells are among the primary anti-tumor immune cells, particularly effector CD8+ T cells (cytotoxic T lymphocytes, CTLs), which can directly recognize and kill tumor cells (14). CTLs recognize antigens presented by MHC-I molecules on the surface of tumor cells (15). Guided by chemokines, CTLs kill tumor cells directly by releasing granules containing granzymes A and B, or by inducing apoptosis through interactions with death ligands, such as Fas ligand (16). CTLs also enhance tumor cell killing by secreting cytokines like interferon-γ (IFN-γ) and tumor necrosis factor-α (TNF-α) (17). Effector T cells: Effector T cells mediate tumor cell apoptosis by secreting cytotoxic molecules such as perforin and granzymes, as well as cytokines like IFN-γ (18). Memory T cells persist after the initial immune response and can rapidly respond upon re-exposure to the same antigen. CD8+ memory T cells provide strong immune protection in the event of tumor recurrence, effectively suppressing the regrowth of tumors (19). CD4+ Helper T Cells (Th Cells): CD4+ T cells promote CTL proliferation and activation by secreting cytokines, enhance the antigen-presenting capability of DCs, and contribute to the formation of memory CTLs, thus playing a key role in long-term anti-tumor immunity (20, 21). DCs: As the most potent antigen-presenting cells (APCs), DCs are critical in initiating adaptive immune responses. They greatly enhance T-cell activation and function by expressing co-stimulatory molecules such as CD80 and CD86, which interact with CD28 on T-cells (22, 23). Additionally, DCs secrete a variety of cytokines (e.g., TNF-α, IL-6, IL-8, and IL-12), which play a key role in regulating local immune responses (24). NK Cells: The absence of MHC-I molecules is a common strategy employed by tumor cells to evade CTL surveillance. NK cells can directly lyse and kill tumor cells lacking MHC-I molecules by recognizing these tumor cells and releasing perforin and granzymes (25). M1 Macrophages: M1 macrophages contribute to the Th1-type immune response by secreting pro-inflammatory cytokines and anti-tumor factors such as TNF-α, IL-1β, and IL-12 (26, 27). They also directly kill tumor cells by releasing reactive oxygen species (ROS) and nitrogen species (RNS) (28).

The synergistic actions of these cells constitute the body’s first line of defense against tumor growth and metastasis. Their activities are extensively regulated by intercellular signaling exchanges within the TIME. Understanding these processes is crucial for developing effective immunotherapeutic strategies against tumors.

### 2.2 Immunosuppressive cells

Regulatory T Cells (Tregs): In the TIME, the substantial enrichment of Tregs suppresses NK cell and CD8+ T cell activity through the massive secretion of immunosuppressive cytokines such as TGF-β, IL-35, and IL-10, which in turn suppress NK cell and CD8+ T cell activity (29, 30). Tregs also induce apoptotic signaling by engaging with death ligands on the surface of effector T cells, such as FasL and PD-L1 (31). In cell contact-dependent effects, Tregs inhibit IL-2 secretion and T-cell activation by blocking CD28-mediated costimulatory signaling via *FBXO38 (* 32). Additionally, Tregs inhibit the antigen-presenting function of DCs by binding LAG-3 protein on their surface to MHC class II molecules on DCs (33, 34). Myeloid-Derived Suppressor Cells (MDSCs): MDSCs inhibit the immune response of effector T cells through multiple mechanisms. They disrupt T cell signaling and metabolic pathways by producing reactive species such as arginase, nitric oxide synthase, and ROS, which degrade intracellular signaling molecules and inhibit cell surface receptor expression (35). MDSCs also diminish T cell proliferation and survival by depleting key immune-stimulating amino acids such as L-arginine. Furthermore, MDSCs inhibit T cell activity by expressing PD-L1 and interacting with Tregs to foster an immunosuppressive environment (36, 37). M2 Macrophages: M2 macrophages suppress inflammation and promote tumor cell survival and proliferation by secreting immunosuppressive factors such as TGF-β and IL-10. They also enhance local immunosuppression by producing chemokines like CCL22, which attract Tregs to the TME (38, 39). M2 macrophages support tumor growth and metastasis by enhancing the tumor cells’ capacity for angiogenesis and tissue remodeling (e.g., through the secretion of VEGF and PDGF) (40).

These immunosuppressive cells interact through intricate mechanisms, forming a complex immunosuppressive network in the TME, which is crucial for tumor growth, progression, and resistance to immunotherapy. Understanding these interactions helps identify new therapeutic targets that may enhance the efficacy of tumor immunotherapy by disrupting these cells’ functions or blocking their signaling pathways.

## 3 Crosstalk between the UPS and various immune cells

### 3.1 T cells

T cells are critical components of anti-tumor immunity, and their normal development and activation are essential for effective immune responses. Recent research has highlighted the pivotal role of immune checkpoints, such as PD-1/PD-L1 and CTLA-4, in enabling tumor immune evasion (41). E3 ubiquitin ligases regulate these specific molecular mechanisms, thereby influencing T cell activity and anti-tumor immune function. These E3 ligases affect the stability, localization, and function of immune checkpoint proteins through specific ubiquitination events (42). FBXO38: FBXO38, a component of the SCF (Skp1-Cullin-F-box) complex, specifically targets the Lys233 residue in the cytoplasmic domain of PD-1 for polyubiquitination (43, 44). This polyubiquitination, typically Lys48-linked, signals for proteasome-dependent degradation of PD-1. Consequently, FBXO38 directly reduces PD-1 expression, inhibiting PD-1/PD-L1 interaction. This reduction significantly enhances T cell activity, thereby improving their cytotoxic effects on tumor cells (45). Cbl-b: Cbl-b inhibits CD28-mediated signaling pathways in T cells. Specifically, Cbl-b actively binds to and ubiquitinates the p85β subunit of PI3K, thereby inhibiting CD28-dependent PI3K pathway signaling (46). CD28 counteracts this by inhibiting Cbl-b’s pathway, with *NEDD4* ubiquitinating Cbl-b under CD28 co-stimulation, leading to Cbl-b’s degradation via the proteasome, thus suppressing its signaling pathway (47, 48). However, within the TME, PD-1 and CTLA4 enhance Cbl-b activity, subsequently inhibiting the expression of Cbl-b downstream effectors and promoting TIME formation. CTLA4, through Cbl-b, inhibits the activity of key proteins such as *PKCθ*, *Vav1*, and *PLCγ*, leading to diminished anti-tumor immune responses by T cells (49). Modulating the ubiquitination status of relevant signaling proteins can therefore enhance T cell activity and improve their tumoricidal capacity. β-TrCP and GSK3β: GSK3β phosphorylates unglycosylated PD-L1, creating a binding site for β-TrCP (50, 51). This phosphorylation is necessary for β-TrCP to mediate the ubiquitination and subsequent degradation of PD-L1 (52). β-TrCP, part of the SCF complex, recognizes and binds phosphorylated PD-L1, leading to its ubiquitination and proteasomal degradation. This reduces PD-L1 levels on the cell surface, thereby diminishing its inhibitory effect on T cells (53). COP9 Signalosome (CSN) and CSN5: CSN5, a component of the COP9 signalosome, functions as a deubiquitinating enzyme that removes ubiquitin chains from PD-L1, preventing its degradation. This leads to sustained high expression of PD-L1 on the cell surface, which in turn enhances the immunosuppressive response (54). Beyond its direct effect on PD-L1, CSN5 modulates the inflammatory response and immune evasion mechanisms by deubiquitinating other key regulatory proteins, such as downstream factors of NF-κB (55, 56).

Tregs play crucial immunosuppressive roles within the TIME, and their functions are directly influenced by the regulation of various ubiquitinating enzymes. Several key deubiquitinating enzymes (DUBs) and E3 ubiquitin ligases are instrumental in regulating Treg stability and function. USP7: FOXP3, a key transcription factor in Treg differentiation, is stabilized and functionally expressed by USP7 (57). USP7 enhances Treg stability and immunosuppressive function by deubiquitinating FOXP3, thereby preventing its degradation. In the TME, this contributes to maintaining Treg-mediated immune suppression, aiding tumor immune evasion (58). USP21: In Tregs, USP21 depletion correlates with a significant reduction in FOXP3 expression and other Treg signature genes, indicating its critical role in maintaining Treg function. Inactivating USP21 may weaken Treg immunosuppression by reducing FOXP3 stability, presenting a potential target for immunotherapy (59). USP22 and USP9X: These DUBs significantly influence T cell activity by regulating PD-L1 deubiquitination (60). Within the TIME, these enzymes help maintain high levels of PD-L1, thereby contributing to tumor-mediated immune suppression and enhancing Treg functionality (61). GRAIL: GRAIL, a RING-type E3 ubiquitin ligase, regulates CD4+ T cell function. Overexpression of GRAIL in Tregs transforms normal CD4+ T cells into regulatory phenotype cells (62). GRAIL also regulates Treg function by degrading the TCR-CD3 complex, thereby reinforcing tumor immune escape. VHL: VHL, another E3 ubiquitin ligase, regulates thymus size and cell number by targeting HIF-1α for ubiquitination and proteasome-dependent degradation (63). Accumulation and enhanced activity of HIF-1α may affect Treg production and function, thereby impacting immune homeostasis in the TME (64). TRAF Family Proteins (TRAF3 and TRAF6): These adaptor E3 ubiquitin ligases regulate T cell and Treg development by influencing IL-2 signaling and thymic stroma development. In mouse models, TRAF3 deletion induces massive Treg proliferation in the thymus, while TRAF6 deletion impedes thymic epithelial cell development, both affecting central tolerance establishment (65).

By elucidating the specific mechanisms of action of these E3 ligases and their impact on T cells, we gain deeper insight into how they regulate tumor immune evasion and T cell activity. These insights provide valuable targets for therapeutic strategies aimed at modulating or interfering with these ubiquitination processes.

### 3.2 DC cells

In the TIME, DC function plays a pivotal role in tumor immunosurveillance and immune escape mechanisms, primarily through the precise regulation of ubiquitination. Ubiquitination modulates DC maturation, antigen-presenting capacity, and interactions with T cells within a complex network of molecular interactions and signal transduction pathways (66). Ubiquitination in DC Maturation and Antigen Presentation: During the immature state of DCs, MARCH1 drives lysosomal degradation and endocytosis by ubiquitinating co-stimulatory molecules like CD86 and the β-subunit of MHCII, thereby maintaining an immune-tolerant state in DCs (67). In the TME, this mechanism may facilitate tumor cells in evading immune surveillance. Upon receiving maturation signals such as TLR agonists, MARCH1 expression is downregulated, reducing the ubiquitin-mediated degradation of MHCII and CD86. This reduction allows these molecules to accumulate on the cell surface, thereby enhancing the DC’s ability to present antigens to T cells and provide co-stimulatory signals essential for activating an anti-tumor immune response (68). Furthermore, CD83 interacts with MARCH1 to inhibit CD86 ubiquitination, promoting stable CD86 expression on the DC surface and enhancing its T cell activation capability (69). This regulatory mechanism is particularly critical in the TIME, as it directly influences the activation state of DCs. Regulation of the NF-κB Pathway by Ubiquitination: NF-κB is a key transcription factor regulating DC function, with its activation tightly controlled by ubiquitination and deubiquitination processes (70). In the TME, inflammatory signals received by DCs, such as TLR agonists, lead to the recruitment of MyD88 and IRAK family kinases, which subsequently interact with the E3 ubiquitin ligase TRAF6 (71). TRAF6, in cooperation with the E2 ligase Ubc13, promotes K63-linked ubiquitination of IRAK1/4, a critical step in activating the IKK complex and NF-κB. During this process, LUBAC (linear ubiquitin chain assembly complex) is crucial for full NF-κB activation (72). LUBAC extends the K63-linked ubiquitin chain through M1-type ligation, which further enhances IKK complex expression and recruitment, ultimately promoting NF-κB release, transcriptional activity, and the pro-inflammatory, activation state of DCs in the TME (73). Ubiquitination in Regulating Tumor Escape Mechanisms: Within the TIME, ubiquitination not only regulates internal signaling and functions of DCs but also influences interactions between tumor cells and DCs by modulating surface molecules on tumor cells (74). MARCH1 is an E3 ligase that can ubiquitinate MHC-II molecules on the surface of tumor cells, leading to their endocytosis and degradation. MHC-II molecules are crucial proteins for antigen presentation, and dendritic cells (DCs) recognize and capture these molecules to present antigens and activate T cells. When MHC-II molecules are degraded by ubiquitin ligases such as MARCH1, the ability of DCs to capture tumor antigens is reduced, thereby suppressing DC function and the anti-tumor immune response (75).

Ubiquitination orchestrates DC function in the TIME through a series of intricate molecular mechanisms. The precise regulation of these mechanisms is vital for activating effective anti-tumor immune responses.

### 3.3 Tumor-associated macrophages

In the TIME, E3 ligases play a crucial role in recruiting TAMs to the tumor site and facilitating their functional expression, thereby promoting tumor growth and immune escape.

CRL: CRL is a large E3 ligase complex composed of multiple subunits, involved in the ubiquitination and degradation of various proteins (76). In the regulation of TAMs, CRL mediates the ubiquitination and subsequent degradation of IκBα, thereby relieving its inhibitory effect on NF-κB. This allows NF-κB to translocate into the nucleus, where it activates the expression of genes associated with cell survival, inflammation, and immune regulation, including CCL2 (77). This process enhances TAM recruitment and their survival within the TME. The activity of CRL is critically dependent on neddylation, a modification of cullin proteins. Inhibition of neddylation leads to inactivation of the CRL E3 ligase complex, reducing the expression of key inflammatory regulators and thereby inhibiting TAM recruitment and tumor cell immune escape (78). COP1 E3 Ligase: COP1 is an E3 ligase with a RING finger domain, responsible for ubiquitinating and degrading several proteins, including the tumor suppressor protein P53 and transcription factors such as C/EBPδ (79). COP1 interacts with C/EBPδ through the adaptor protein Trib, promoting the ubiquitination and degradation of C/EBPδ. In many tumor cells, C/EBPδ functions to inhibit the expression of chemokines and chemotactic factors (80). The degradation of C/EBPδ enhances the secretion of chemokines, such as CCL2, by TAMs, thereby increasing TAM recruitment. Modulating COP1 activity can influence the polarization and function of TAMs towards the M2 phenotype, which is crucial for reinforcing immunosuppression within the TIME (81). KCP1 E3 Ligase: KCP1 mediates the ubiquitination and conversion of p105, the precursor of the NF-κB protein, into the active p50 subunit. p50 is a key regulator of the NF-κB pathway, which governs the expression of a broad range of genes involved in inflammatory responses and immunity (82, 83). Overexpression of p50 has been shown to promote the expression of chemokines such as CCL3 and CCL4, which are vital mediators of TAM recruitment. Additionally, the p50 subunit influences PD-L1 expression, and its downregulation may enhance anti-tumor immune responses within the TIME (84). In tumors, loss of function or downregulation of KCP1 has been observed, which may disrupt the NF-κB signaling pathway and alter the TIME.

**Figure 1:**
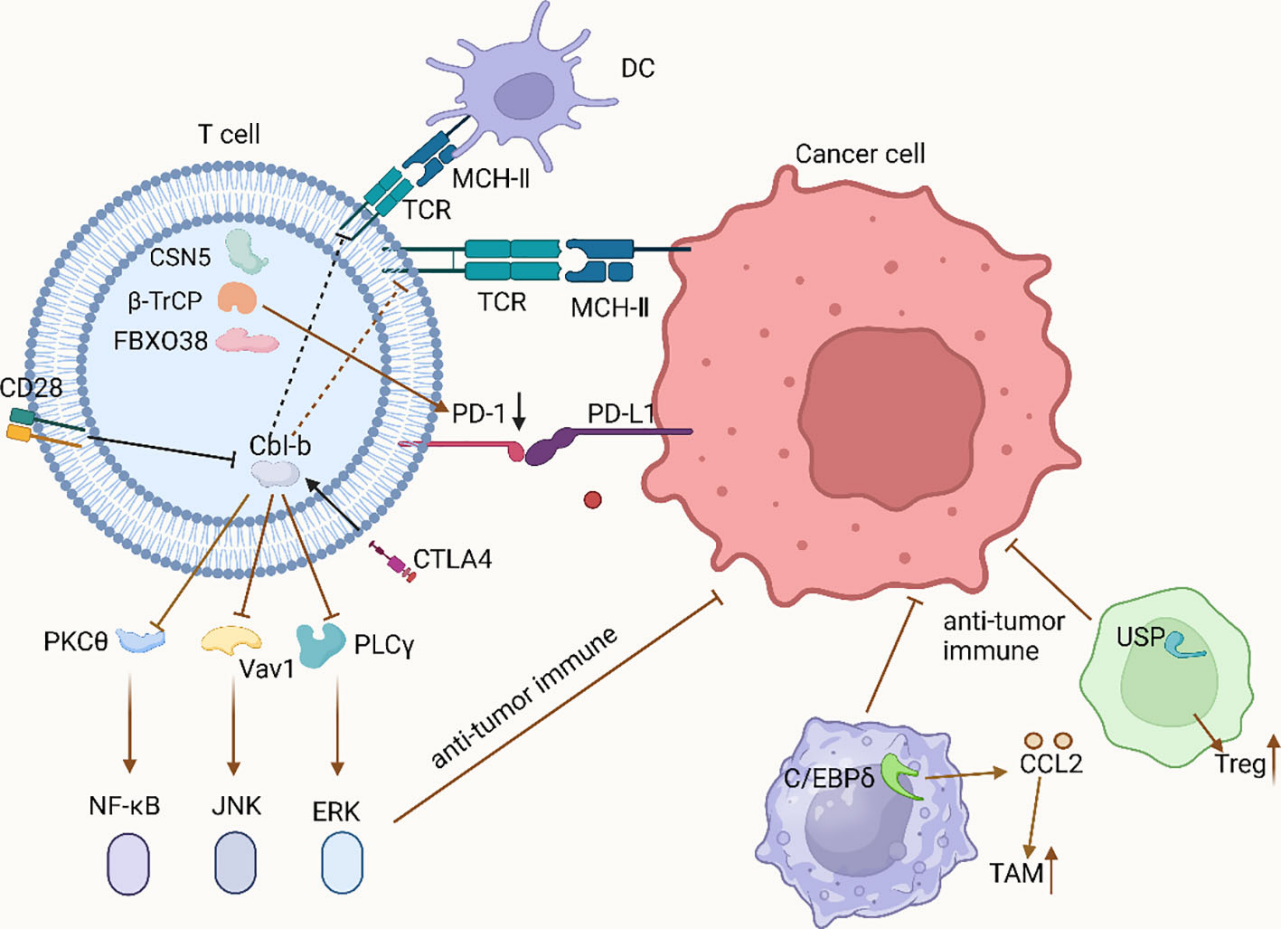
UPS regulates various types of immune cells in TIME.

The UPS in the TIME influences the growth, activation, and functional expression of various immune cells by precisely regulating the stability and activity of key proteins (**Figure 1**). Targeting these mechanisms has led to several new clinical trials with promising outcomes (**Table 1**). In the future, focusing on the interaction between the UPS and immune cells is expected to provide a robust theoretical foundation for developing novel combination therapies against tumors.

**Table 1:** Clinical trials related to ubiquitinating enzymes in tumor therapy.

NCT Number	Title	Status	Conditions	Interventions	Characteristics
NCT06223542	Studying TAK-243 in Patients With Advanced Cancer	Not yet recruiting	• Advanced Lymphoma • Advanced Malignant Solid Neoplasm • Metastatic Malignant Solid Neoplasm	• Procedure: Biopsy • Procedure: Biospecimen Collection • Procedure: Computed Tomography • Procedure: Magnetic Resonance Imaging • Drug: UAE Inhibitor TAK-243	Phase: Phase 1
NCT05107674	A Study of NX-1607 in Adults With Advanced Malignancies	Recruiting	• Ovarian Cancer, Epithelial • Gastric Cancer • GastroEsophageal Junction (GEJ) Cancer • Head and Neck Squamous Cell Carcinoma • Metastatic or Unresectable Melanoma • Non-small Cell Lung Cancer (NSCLC) • Metastatic Castration-resistant Prostate Cancer (mCRPC) • Malignant Pleural Mesothelioma (MPM) • Triple Negative Breast Cancer (TNBC) • Metastatic Urothelial Carcinoma • and 4 more	• Drug: NX-1607 • Drug: Paclitaxel	Phase: Phase 1
NCT02256241	The Role of RING Ubiquitin Ligases in Biologic and Oncologic Processes in Tissues of Mesenchymal Origin	Unknown status	• Group 1: Trauma Operation for Otherwise Healthy Patients • Group 2: Primary Tumors of Mesenchymal Origin		
NCT02045095	A Dose Escalation Study of MLN7243 (TAK-243) in Adult Participants With Advanced Solid Tumors	Terminated	• Advanced Malignant Solid Tumors	• Drug: MLN7243	Phase: Phase 1
NCT01358617	Prognostic Biomarkers in Tumor Tissue Samples From Young Patients With Neuroblastoma	Completed	• Disseminated Neuroblastoma • Localized Resectable Neuroblastoma • Localized Unresectable Neuroblastoma • Recurrent Neuroblastoma • Stage 4S Neuroblastoma	• Other: diagnostic laboratory biomarker analysis	
NCT00216697	An Extension Study to Provide Bortezomib to Patients With Relapsed or Refractory Multiple Myeloma Who Previously Participated in a Bortezomib Phase I/II Study and Who May Benefit From Re-Treatment With or Continuation of Bortezomib Therapy	Completed	• Multiple Myeloma	• Drug: bortezomib	Phase: Phase 2

## 4 Clinical applications of the UPS in tumor therapy, challenges and perspectives

### 4.1 The role of E3 ligase inhibitors in immunotherapy

Inhibitors targeting E3 ligases have demonstrated significant potential in cancer therapy. SPOP is an E3 ligase that ubiquitinates and degrades PD-L1 protein, thereby inhibiting tumorigenesis. Studies have shown that CDK4/6 can inhibit the expression of Cyclin D-CDK4, leading to the phosphorylation of Speckle-type POZ protein (SPOP), which ultimately stabilizes PD-L1 expression and increases its protein levels (85). Therefore, CDK4/6 inhibitors can enhance the ubiquitination function of SPOP, promoting anti-tumor immune responses. Research by Pan et al. has confirmed that CDK4/6 inhibitors, when combined with anti-PD-1 inhibitors, significantly inhibit tumor growth and proliferation in mice, resulting in a marked increase in overall survival.

ARV-471 is an orally administered drug that utilizes PROTAC technology to target estrogen receptor (ER) α. In mouse models of breast cancer, the combination of ARV-471 with the CDK4/6 inhibitor Palbociclib significantly suppressed tumor progression (86). Additionally, FBXO22 is a typical F-box protein and a component of E3 ligase complexes. Similar to the action of SPOP, FBXO22 directly ubiquitinates and degrades PD-L1, thereby increasing the sensitivity of tumor cells to immunotherapeutic agents. Among the upstream regulators of FBXO22, CDK5 is particularly noteworthy (87). CDK5 directly influences the ubiquitination and degradation of PD-L1, thereby modulating anti-tumor immune responses. Studies have shown that combining CDK5 inhibitors with immune checkpoint inhibitors (ICIs) can significantly enhance the efficacy of immunotherapy, effectively inhibiting tumor initiation and progression.

### 4.2 Clinical applications of DUB inhibitors in immunotherapy

DUBs are primarily responsible for removing ubiquitin molecules from protein substrates, thereby reversing ubiquitination modifications. This process is crucial for maintaining protein homeostasis within the cell, regulating signal transduction pathways, and modulating various cellular functions. DUBs play an essential role in maintaining immune homeostasis and modulating immune responses by regulating the ubiquitination status of key immune-related proteins (88). For example, DUBs remove ubiquitin chains from PD-L1, preventing its degradation by the proteasome and thereby maintaining high levels of PD-L1 expression on the surface of tumor cells. This elevated PD-L1 expression binds to PD-1 on T cells, inhibiting their activity and leading to immune evasion. DUBs, such as USP7, have been found to enhance the stability and function of Tregs by deubiquitinating FOXP3. Tregs exert immunosuppressive effects in the TME, limiting the anti-tumor activity of effector T cells. Targeting DUBs to modulate their activity has emerged as a potential strategy to enhance anti-tumor immune responses and inhibit tumor progression. DUB inhibitors are of great significance in the clinical treatment of tumors. Up to now, several clinical trials have made some progress. A clinical trial, NCT02372240, showed that VLX1570, as the first DUB inhibitor to enter a clinical trial, was effective in killing tumors by mainly targeting USP14/UCH-L5. However, unfortunately, the clinical trial was terminated due to severe pulmonary toxicity (89). KSQ-4279, as this most advanced USP1 inhibitor reported so far, is undergoing phase I clinical trials (NCT05240898). It is primarily used for the treatment of advanced solid tumors, including ovarian and triple-negative breast cancers. KSQ-4279 exhibits excellent pharmacokinetic properties *in vitro* and shows significant antitumor activity in animal models. In particular, when combined with PARP inhibitors, it was able to kill tumors in multiple models, demonstrating in addition to excellent clinical efficacy (90). In addition, the first-generation USP7 inhibitor P5091 induces apoptosis in multiple myeloma cells by promoting ubiquitination of MDM2 and MDMX, which in turn activates the p53 pathway and ultimately induces apoptosis. It has achieved good efficacy in the clinical treatment of myeloma. The second-generation covalently bound inhibitor P22077 inhibits the enzymatic activity of USP7 by modifying cysteine 223 in its catalytic center. It has gained some efficacy in the treatment of many tumors (91). However, since there are still obvious side effects, its therapeutic mechanism still needs to be further explored in the future. The natural product berberine, as a novel USP7 inhibitor, promotes ubiquitination and degradation of MDM2 by disrupting the MDM2-DAXX-USP7 complex, and has also achieved good efficacy in the clinical treatment of tumors with fewer side effects. Other DUB inhibitors, such as USP21, USP14 and OTUB2, have also shown great therapeutic potential in a variety of solid tumors. USP21 inhibitors are able to inhibit the deubiquitylation of MEK2 and FOXM1, which in turn down-regulates the expression of the ERK pathway and ultimately inhibits the growth of tumor cells (92). Despite the challenges of toxicity, targeting, and drug resistance, DUB inhibitors will be a key component in the clinical treatment of cancer in the future.

USP7 enhances the stability and function of Tregs by deubiquitinating FOXP3. Tregs play an immunosuppressive role in the TME, limiting the anti-tumor activity of effector T cells. Additionally, studies have found a significant positive correlation between the expression of USP7 and PD-L1. Further research revealed that USP7 can assist tumor cells in immune evasion by increasing the expression of PD-L1 protein. The combination of USP7 inhibitors with PD-1 or PD-L1 inhibitors can significantly enhance the efficacy of immunotherapy. Moreover, combining USP7 inhibitors with anti-PD-1 monoclonal antibody therapy has shown promising therapeutic effects in lung cancer models (93). USP22 also has the ability to regulate PD-L1 stability through deubiquitination. USP22 increases PD-L1 protein levels by regulating the CSN5/PD-L1 axis. CSN5 has been confirmed as a key protein that promotes the deubiquitination of PD-L1. USP22 stabilizes CSN5 protein by deubiquitinating polyubiquitin chains, thereby enhancing PD-L1 expression. Knocking down USP22 can enhance T cell and NK cell activity, and when combined with ICIs, it significantly improves the therapeutic efficacy of ICIs (94). USP14 promotes IDO1 expression by enhancing its deubiquitination, preventing its degradation by ubiquitin ligases. High expression of IDO1 protein significantly suppresses CD8+ T cell activity and levels, facilitating immune evasion. In colorectal cancer (CRC), knocking down USP14 can inhibit IDO1 expression, enhance CD8+ T cell activity and numbers, and make CRC cells more sensitive to immunotherapy. Furthermore, clinical studies have shown that the first-generation USP14 inhibitor IU1 can significantly reduce IDO1 protein expression and inhibit IDO1-induced immune suppression. Additionally, the combination of IU1 with ICIs and IDO1 inhibitors can significantly reduce the “off-target” effects associated with these inhibitors and enhance therapeutic efficacy (95). The combined treatment of IU1 and anti-PD-1 has been shown to significantly reduce tumor proliferation and progression, offering a new therapeutic approach for future cancer patients.

### 4.3 Challenges and perspectives

USP7, USP22, and USP14 remain pivotal targets for the combination of UPS inhibitors and immunotherapy. In addition, USP8, USP15, USP9X, and USP18 can directly bind to PD-L1, stabilizing PD-L1 expression and promoting immune evasion by tumor cells. Inhibitors targeting these USPs, when combined with ICIs, can significantly enhance the efficacy of immunotherapy. However, PD-1/PD-L1 regulation is not solely dependent on ubiquitination; other post-translational modifications (PTMs), such as phosphorylation, acetylation, lactylation, and palmitoylation, also play critical roles in modulating PD-1/PD-L1 protein expression. Future research should, therefore, take a comprehensive approach to protein degradation pathways and explore multi-target combination therapies. Despite the immense potential of DUB inhibitors in the clinical treatment of malignant tumors, several challenges remain in developing specific inhibitors. First, the complex structural features of DUB catalytic domains, coupled with the high similarity among DUB family members, present significant challenges for targeted drug design. Second, the large molecular weight of DUBs complicates crystal formation, making it difficult to obtain complete crystal structures—an essential requirement for structure-based drug design. Additionally, DUBs may undergo conformational changes upon ubiquitin binding, which further complicates small molecule prediction and computer simulation. Moreover, the intricate regulatory mechanisms of DUBs, which involve both catalytic activity and substrate-mediated conformational modulation, add another layer of complexity to the development of specific inhibitors. Finally, given the critical role of the UPS in normal cellular functions, inhibiting UPS components may lead to severe toxicity and side effects, such as peripheral neuropathy and hematologic toxicity observed with proteasome inhibitors.

Nevertheless, the potential clinical application of DUB inhibitors in cancer treatment remains promising. Future research could focus on identifying and optimizing novel small-molecule DUB inhibitors through high-throughput screening and computer-aided drug design (CADD). Advances in structural biology techniques, such as cryo-electron microscopy (Cryo-EM), may provide clearer crystal structures of DUBs, thereby facilitating the rational design of inhibitors. Additionally, developing multi-target inhibitors that can simultaneously target multiple DUBs may enhance therapeutic efficacy and reduce tumor drug resistance by inhibiting multiple signaling pathways concurrently. The combined use of DUB inhibitors with immunotherapy, chemotherapy, and other therapeutic modalities could significantly improve cancer treatment outcomes. The integration of genomics, proteomics, and metabolomics technologies, alongside a deeper understanding of the specific mechanisms of DUBs in various cancers, will offer more personalized treatment options for patients. Ultimately, the continued development and clinical application of DUB inhibitors hold the potential to substantially improve the survival rates and quality of life for cancer patients.

## 5 Conclusion

The ubiquitin-proteasome system regulates immune system responses by interacting with various types of immune cells in TIME. In this review, we aim to summarize the essential components of TIME, focusing on the normal function of T cells, DC cells, NK cells, MDSC, M2-type macrophages, and Tregs. UPS plays an important role in facilitating immune evasion by modulating these immune cells. crucial role in helping tumor cell immune evasion by regulating these immune cells. In the future, targeting relevant ubiquitinating enzymes and combining them with immunotherapy will greatly promote the efficacy of tumor therapy and significantly improve the quality of patient’s survival.

## Glossary

UPS: Ubiquitin-Proteasome System
TIME: Tumor Immunosuppressive Microenvironment
TME: Tumor Microenvironment
PD-1: Programmed Cell Death Protein 1
PD-L1: Programmed Death-Ligand 1
CTLA-4: Cytotoxic T-Lymphocyte-Associated Protein 4
SCF: Skp1-Cullin-F-box
PI3K: Phosphoinositide 3-Kinase
NF-κB: Nuclear Factor kappa-light-chain-enhancer of activated B cells
β-TrCP: Beta-Transducin Repeat Containing Protein
GSK3β: Glycogen Synthase Kinase 3 Beta
COP9: Constitutive Photomorphogenesis 9
CSN: COP9 Signalosome
DUBs: Deubiquitinating Enzymes
USP7: Ubiquitin-Specific Protease 7
USP21: Ubiquitin-Specific Protease 21
USP22: Ubiquitin-Specific Protease 22
USP9X: Ubiquitin-Specific Protease 9 X-Linked
GRAIL: Gene Related to Anergy in Lymphocytes
TCR: T-Cell Receptor
CD3: Cluster of Differentiation 3
VHL: Von Hippel-Lindau Tumor Suppressor
HIF-1α: Hypoxia-Inducible Factor 1-alpha
TRAF: TNF Receptor Associated Factor
MARCH1: Membrane-Associated RING-CH-type Finger 1
MHCII: Major Histocompatibility Complex Class II
CD86: Cluster of Differentiation 86
TLR: Toll-Like Receptor
MyD88: Myeloid Differentiation Primary Response 88
IRAK: Interleukin-1 Receptor-Associated Kinase
IKK: IκB Kinase
LUBAC: Linear Ubiquitin Chain Assembly Complex
CRL: Cullin-RING Ligase
IκBα: Inhibitor of kappa B alpha
C/EBPδ: CCAAT/Enhancer-Binding Protein Delta
KCP1: Kelch-Like ECH-Associated Protein 1
p105: Precursor of NF-κB Protein
p50: Active Subunit of NF-κB Protein
DC: Dendritic Cell
Tregs: Regulatory T Cells
Th17: T Helper 17 Cells
CTL: Cytotoxic T Lymphocytes
APC: Antigen-Presenting Cells
TNF-α: Tumor Necrosis Factor Alpha
IL-6: Interleukin 6
IL-8: Interleukin 8
IL-12: Interleukin 12
CCL2: C-C Motif Chemokine Ligand 2
CCL3: C-C Motif Chemokine Ligand 3
CCL4: C-C Motif Chemokine Ligand 4
TGF-β: Transforming Growth Factor Beta
IL-10: Interleukin 10
IL-35: Interleukin 35
VEGF: Vascular Endothelial Growth Factor
PDGF: Platelet-Derived Growth Factor
LAG-3: Lymphocyte-Activation Gene 3
